# Case report: Extraskeletal Ewing sarcoma with a germline pathogenic variant of *SMARCA4*

**DOI:** 10.3389/fonc.2024.1422605

**Published:** 2024-10-08

**Authors:** Min-Chae Kang, Sun-Young Kong, Sang-Yoon Park, Seog-Yun Park, Eun-Gyeong Lee, Chong Woo Yoo, Yun Hwan Kim, Hyeji Kim, Wonyoung Choi

**Affiliations:** ^1^ Division of Rare and Refractory Cancer, National Cancer Center, Goyang, Republic of Korea; ^2^ Department of Cancer Biomedical Science, National Cancer Center Graduate School of Cancer Science and Policy, Goyang, Republic of Korea; ^3^ Department of Laboratory Medicine, National Cancer Center, Goyang, Republic of Korea; ^4^ Center for Gynecologic Cancer, Research Institute and Hospital, National Cancer Center, Goyang, Republic of Korea; ^5^ Department of Pathology, National Cancer Center, Goyang, Republic of Korea; ^6^ Center for Breast Cancer, National Cancer Center, Goyang, Republic of Korea; ^7^ Research Institute and Hospital, National Cancer Center, Goyang, Republic of Korea; ^8^ Department of Obstetrics and Gynecology, Ewha Womans University Mokdong Hospital, Seoul, Republic of Korea; ^9^ Division of Cancer Biology, National Cancer Center, Goyang, Republic of Korea

**Keywords:** ovarian cancer, Ewing sarcoma, genetic counseling, SMARCA4 mutation, Next-generation sequence (NGS)

## Abstract

*SMARCA4* (BRG1) is a core unit of the SWI/SNF complex, regulating gene transcription through chromatin remodeling. Germline *SMARCA4* variants have been reported to be associated with various malignancies. Here, we report the first case of extraskeletal Ewing sarcoma in a young female patient with a germline pathogenic variant of *SMARCA4* (c.3546 + 1G>A), diagnosed with next generation sequencing (NGS). This alteration was also identified in her familial lineage, including her sister who was previously diagnosed with small cell carcinoma of the ovary, hypercalcemic type, a malignancy highly associated with *SMARCA4* mutations. Despite undergoing radical surgery and receiving systemic treatments including VeIP (vinblastine, ifosfamide, cisplatin), and VDC (vincristine, doxorubicin, cyclophosphamide) regimens, the patient succumbed to death due to disease progression. With the implementation of NGS, we anticipate that more cases with *SMARCA4* mutations will be diagnosed in the future. Further research is necessary to unveil therapeutic targets associated for this oncogenic alteration.

## Introduction

The switch/sucrose non-fermenting (SWI/SNF) complex regulates gene transcription through chromatin remodeling, playing a pivotal role in the modulation of gene expression (1). Given its significant function, subunits of the SWI/SNF complex have been identified as tumor suppressors in human cancer (2–5). Indeed, the SWI/SNF complex emerges as the most commonly mutated chromatin-regulatory complex human cancers, with alterations identified in approximately 20% of all cases (6). *SMARCA4*, located on chromosome arm 19p, is an essential component of the SWI/SNF complex, encoding the transcriptional activator protein BRG1. Through its ATPase activity, BRG1 has the capability to both activate or repress transcription (7). Inactivation of *SMARCA4* is associated with various cancer types such as lung, brain, ovarian, and pancreatic cancer (8–11). Particularly, *SMARCA4* variants, including both germline (43%) and somatic (57%) mutations, have been implicated as causative genetic alterations in small cell carcinoma of the ovary, hypercalcemic type (SCCOHT) (12).

Ewing sarcoma (ES) represents a category of small round cell tumors predominantly arising in bone, ranking as the second most prevalent bone malignancy among children and young adults (13). Extraskeletal Ewing sarcoma (EES) is exceptionally rare, with no documented cases previously reported in individuals with a germline pathogenic variant in *SMARCA4*. Here, we present the first documented case of EES affecting the ovary in a young female patient harboring a germline pathogenic variant in *SMARCA4*.

## Case report

**Figure 1:**
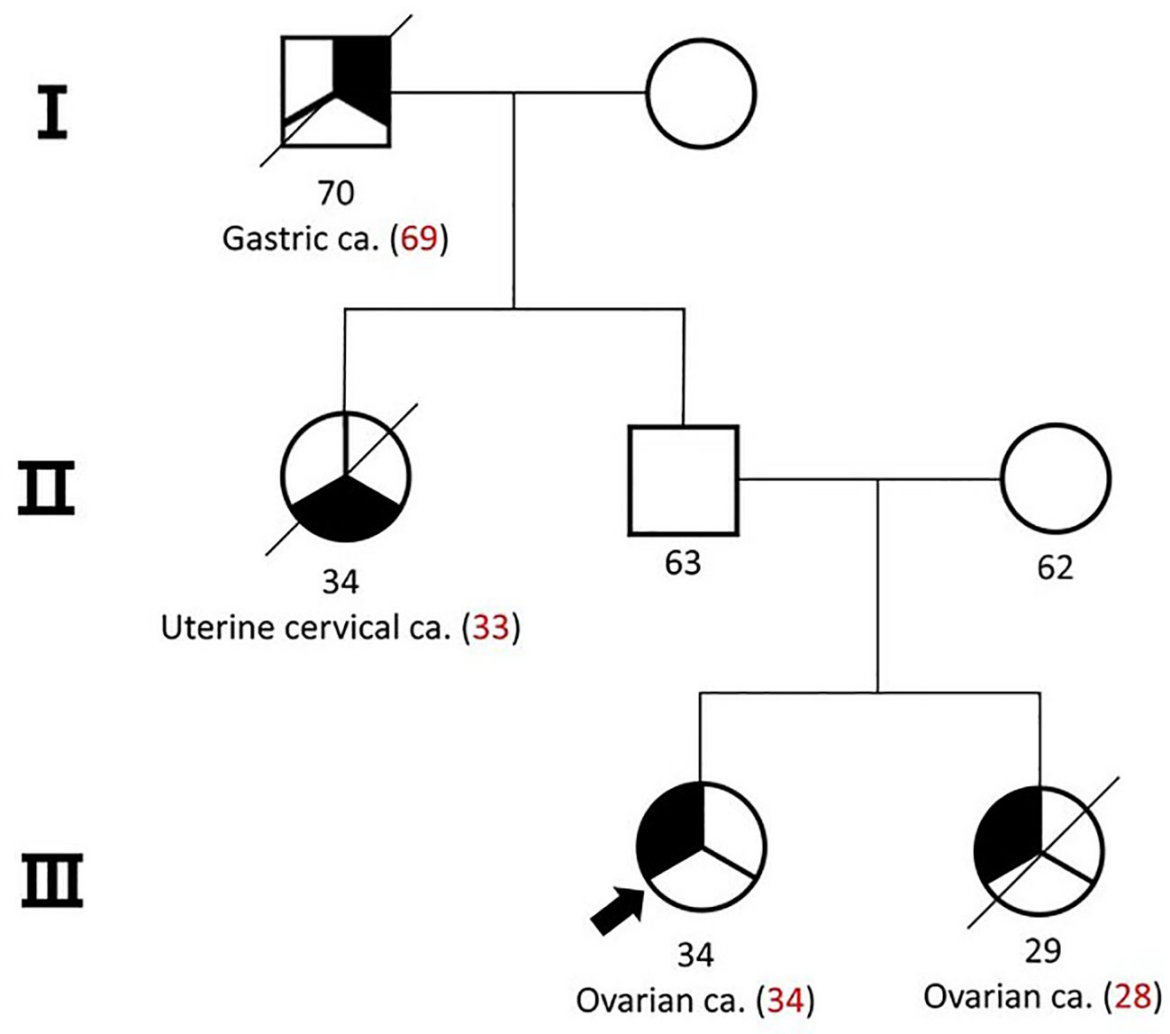
Pedigree of the patient and her family. Her sister was diagnosed with small cell carcinoma of the ovary-hypercalcemic type (SCCOHT).

A 34-year-old female patient was referred to our institute with a 7.8 cm sized mass in her left ovary. She had a familial history of ovarian cancer in a first-degree relative and gastric and cervical cancers in second-degree relatives (**Figure 1**). Initial computed tomography (CT) scans revealed an enlarged paraaortic lymph node suggestive of metastatic involvement. Serum tumor marker analysis revealed a slight elevation in carbohydrate antigen 125 (CA125, 36.1 U/ml; reference range, 0-35 U/ml), while levels of carcinoembryonic antigen (CEA), carbohydrate antigen 19-9 (CA 19-9), alpha-fetoprotein (AFP), and beta-human chorionic gonadotropin (b-HCG) remained within normal limits.

**Figure 2:**
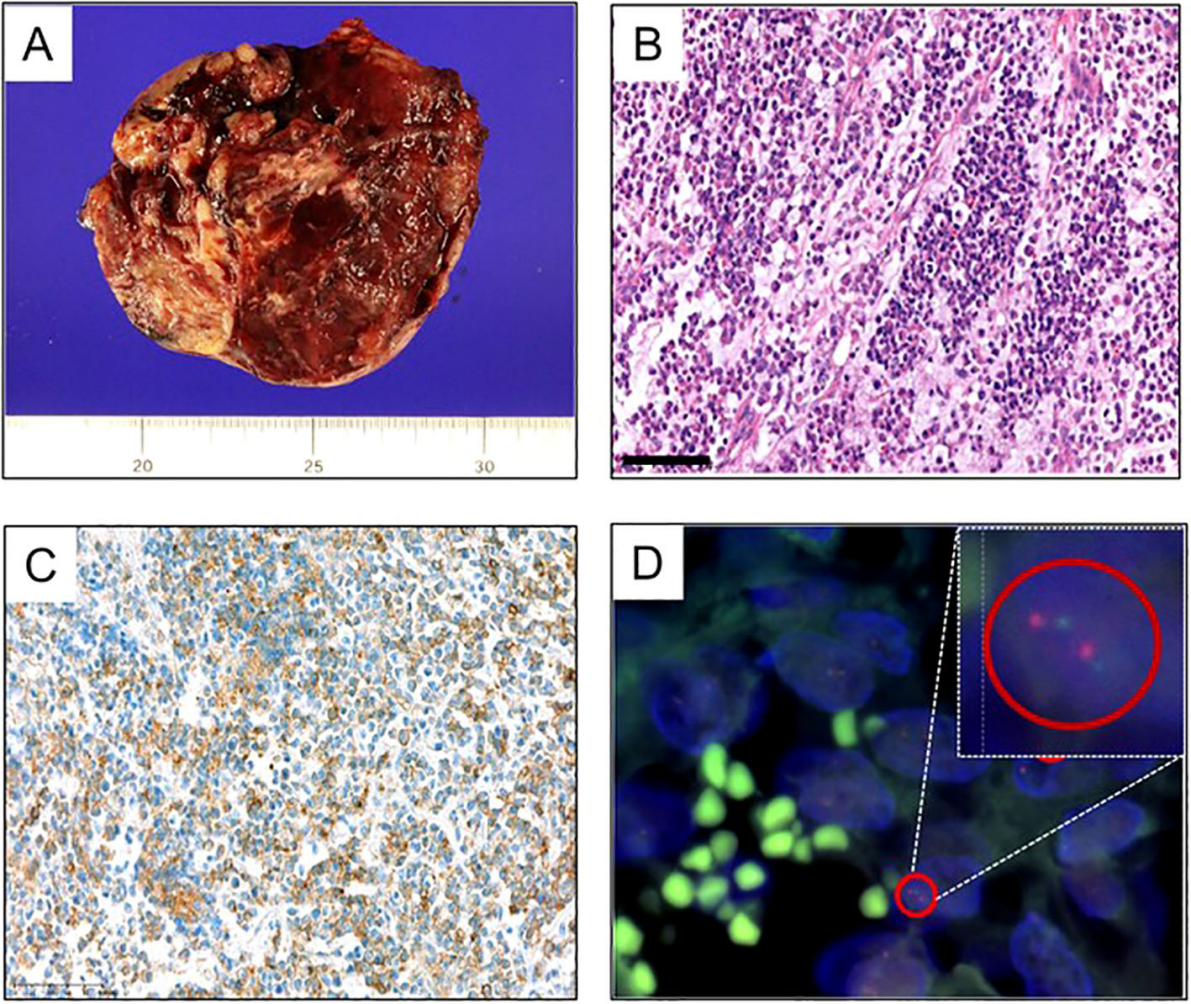
Pathologic features of the patient’s tumor. **(A)** Gross tumor resected from the initial cytoreductive surgery of ovary. **(B–D)** Results of the resected tumor from the second cytoreductive surgery. **(B)** H&E staining of the resected tumor, suggestive of malignant stromal cell tumor with poor differentiation (200x). **(C)** Immunohistochemical staining for CD99 represented positive. **(D)** Fluoresent *in-situ* hybridization for EWSR1 confirmed the EWSR1 gene translocation.

Subsequent to the referral, the patient underwent cytoreductive surgery, encompassing a total abdominal hysterectomy, bilateral salpingo-oophorectomy, omentectomy, appendectomy, and aortic, and pelvic lymph node biopsy (PLNB) (**Figure 2A**). The excised tumor from the left ovary, measuring 9.2 x 8.2 cm, demonstrated capsular invasion without evidence of regional lymph node metastasis. Pathological examination initially characterized the tumor as a malignant stromal cell tumor with poor differentiation, suggestive of a Sertoli-Leydig cell tumor. This characterization was supported by immunohistochemical staining results, which showed negative reactions for PAX8 (Paired box gene 8), Inhibin, AFP, and Sall4, but focal positivity for Calretinin and CA125.

In the absence of residual tumor on postoperative CT scans, the patient was administered 5 cycles of adjuvant chemotherapy, consisting of bleomycin, etoposide, and cisplatin (BEP). However, three months after the final dose of chemotherapy, CT scans revealed new tumor lesions within the pelvic cavity and enlargement of multiple paraaortic and pelvic lymph nodes. A second cytoreductive surgery was performed, revealing pathology findings consistent with those of a malignant stromal cell tumor. The immunohistochemical profile closely matched that of the initial tumor; however, positive staining for CD99 and confirmation of the EWSR1 gene translocation through fluorescent *in-situ* hybridization (FISH) supported a diagnosis of Ewing sarcoma (**Figures 2B–D**).

**Figure 3:**
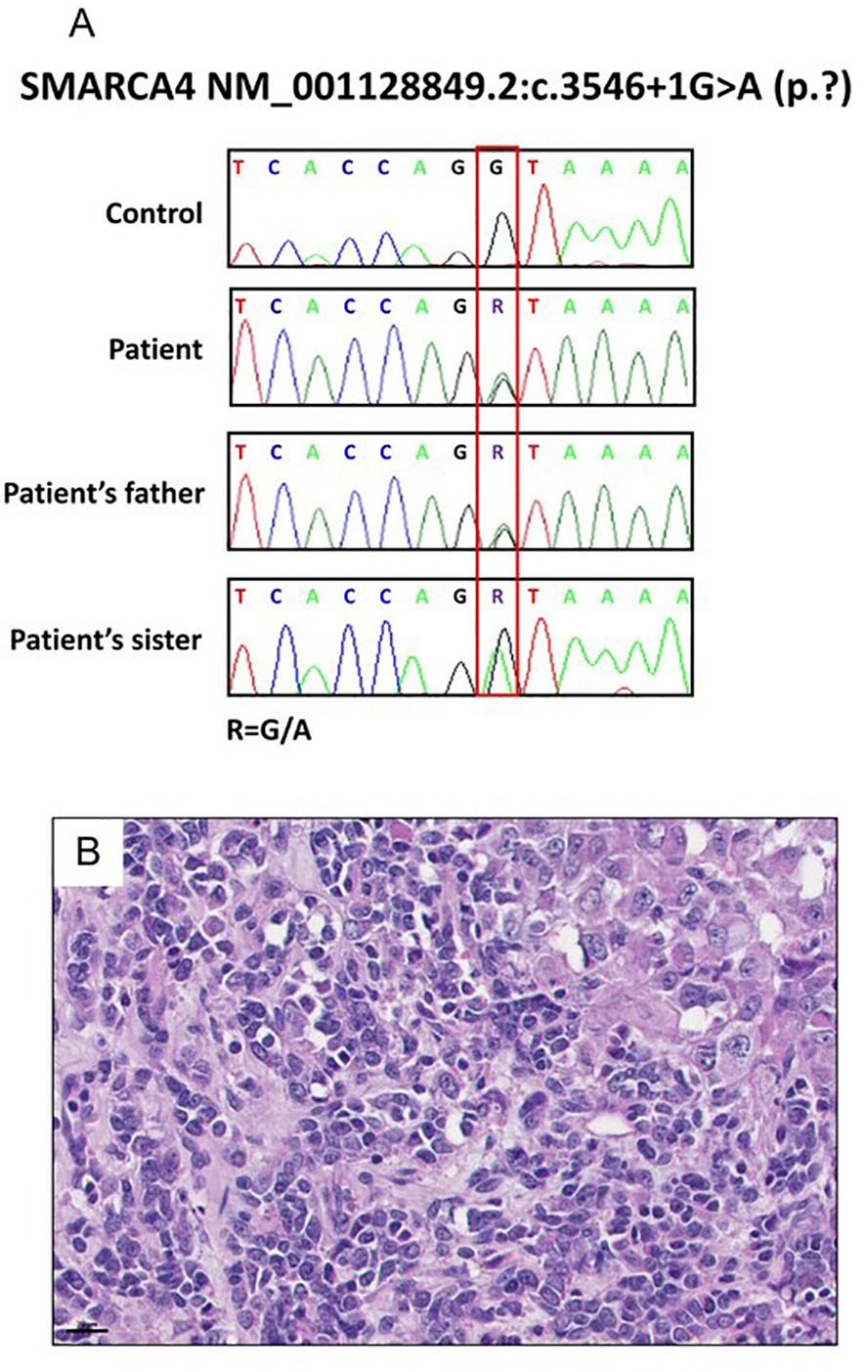
Results of the patient’s family specimen. **(A)** *SMARCA4* c.3546 + 1G>A was confirmed in a patient and her sister and father by Sanger sequencing. **(B)** H&E staining of the sister’s resected tumor suggestive of small cell carcinomas of the ovary, hypercalcemic type (SCCOHT) (40x).

Due to her young age at diagnosis and familial cancer history, a comprehensive next-generation sequencing (NGS) panel test encompassing 73 genes was conducted to identify germline mutations (**Supplementary Table S1**). This analysis identified a pathogenic intron variant in *SMARCA4* (c.3546 + 1G>A), a mutation that was also observed in her father later (**Figure 3A**). Notably, her sister had been previously diagnosed with small cell carcinoma of the ovary, hypercalcemic type (SCCOHT) (**Figure 3B**), a condition closely associated with germline *SMARCA4* mutations, although no genetic testing had been performed at the time of her diagnosis. We obtained the patient’s sister’s tumor tissue, and Sanger sequencing confirmed the presence of the same *SMARCA4* pathogenic variant in her tissue sample (**Figure 3A**).

**Figure 4:**
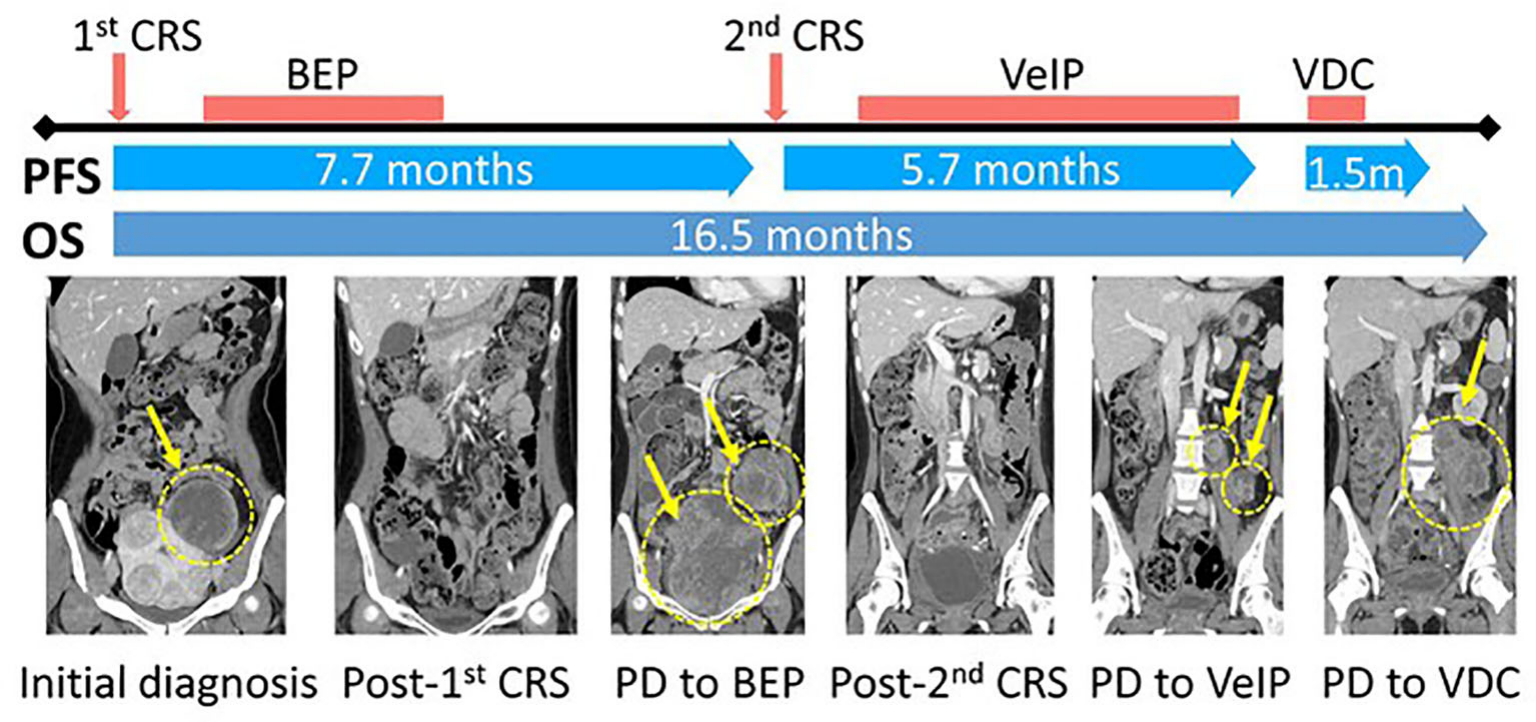
Schematic diagram of the clinical course and representative CT images of the tumor. CRS, Cytoreductive surgery; PFS, Progression-free survival; OS, Overall survival; BEP, (Bleomycin, Etoposide, and Cisplatin); VeIP, (Vinblastine, Ifosfamide, Cisplatin); VDC, (Vincristine, Doxorubicin, Cyclophosphamide).

Based on the revised histological diagnosis of extraskeletal Ewing sarcoma, she underwent subsequent chemotherapy consisting of vinblastine, ifosfamide, and cisplatin (VeIP) for 7 cycles (5 months), followed by vincristine, doxorubicin, and cyclophosphamide (VDC) for 2 cycles (1 month) (**Figure 4**). However, despite these systemic treatments, the patient’s condition continued to deteriorate, and she succumbed to death due to disease progression 17 months after the initial diagnosis.

## Discussion

Germline mutations in *SMARCA4* have been implicated in a range of malignancies, including rhabdoid tumor predisposition syndrome 2 (RTPS2) and SCCOHT (7). Additionally, multiple studies have documented associations between mesenchymal malignancies and either germline or somatic alterations in *SMARCA4* (14, 15), However, this represents the first reported case of extraskeletal Ewing sarcoma manifesting in association with a germline *SMARCA4* mutation.

Ewing sarcoma is a highly rare and aggressive type of mesenchymal tumor, with a 5-year overall survive rate of approximately 70**–**80% for localized disease, while it drops to 15**–**30% for metastatic disease (16, 17). Its pathologic diagnosis molecularly defined by the presence of fusions between EWSR1 or FUS genes and one of the members of the ETS family of transcription factor genes (18). Moreover, Ewing sarcoma is notably characterized by its distinctive expression of CD99, which usually exhibits a diffuse and strong membranous pattern. While the majority of Ewing sarcoma cases present as primary bone tumors, the occurrence of extraskeletal Ewing sarcoma is particularly rare. Owing to its infrequency, there is a limited number of studies focused on investigating the most effective treatment strategies and clinical outcomes for extraskeletal Ewing sarcoma. However, a retrospective analysis utilizing a comprehensive database indicated that the prognosis for patients with extraskeletal Ewing sarcoma does not significantly deviate from those diagnosed with bone Ewing sarcoma (19). Consequently, current clinical guidelines advocate for the application of a uniform therapeutic strategy across all variants of Ewing sarcoma (20).

Recent advancements in molecular and clinicopathological research have led to the proposal of a new classification for undifferentiated thoracic malignancies, identifying *SMARCA4* inactivation as a distinct disease entity (21, 22). Remarkably, unsupervised hierarchical clustering of transcriptome data has revealed that samples of *SMARCA4*-deficient thoracic sarcoma (*SMARCA4*-DTS) tend to cluster alongside SCCOHT, and malignant rhabdoid tumors (MRT), which are the representative malignancies linked to germline *SMARCA4* aberrations (21). Furthermore, the pathological features of *SMARCA4*-DTS, marked by the presence of discohesive cells with pronounced nucleoli, strikingly mirror the characteristics described for SCCOHT, as well as those observed in our case patient (23). Moreover, the typically poor clinical outcomes associated with *SMARCA4*-DTS and SCCOHT align with the limited response to systemic treatment and the aggressive tumor behavior observed in this case patient. Although a larger dataset of molecular and pathological data is necessary for a conclusive determination, these observations strongly imply that *SMARCA4* deficiency might act as a significant oncogenic driver, potentially leading to a highly aggressive cancer phenotype, regardless of its anatomical origin.

The exploration of *SMARCA4* deficiency as a therapeutic target has recently garnered interest. Loss-of-function alterations in the subunits of the SWI/SNF complex have been demonstrated to augment the activity of the polycomb repressive complex 2 (PRC2) signaling pathway, leading to substantial chromatin remodeling (24). EZH2, serving as the catalytic subunit of the PRC2 complex, represents a pharmacological target for tazemetostat. In a recent basket study, patients whose tumors exhibited EZH2 mutations or demonstrated loss of SMARCB1 or SMARCA4, as identified through immunohistochemical staining, were selected to receive tazemetostat treatment (25). In the trial comprising 20 patients, one individual diagnosed with non-Langerhans cell histiocytosis showed a loss of SMARCA4 expression. Although the primary endpoint of overall response rate was not achieved—with only one patient showing a partial response—it is crucial to emphasize that the patient with the lost SMARCA4 expression was the one who responded to the treatment. This patient received tazemetostat for 26 cycles, equating to a treatment duration of 2 years. Ongoing clinical trials are also investigating novel therapeutic options. A phase I/II trial is currently testing the combination of tazemetostat with nivolumab (anti-PD1) and ipilimumab (anti-CTLA4) in patients with SMARCA4 loss confirmed by immunohistochemical staining or molecular studies (ClinicalTrials.gov ID NCT05407441). Additionally, a novel SMARCA4/SMARCA2 inhibitor, FHD-286, is being evaluated in a phase 1 dose escalation study in patients with advanced hematologic malignancies (ClinicalTrials.gov ID NCT04891757). While this study does require confirmation of SMARCA4 alterations for inclusion, its findings on clinical efficacy in advanced hematologic malignancies could offer valuable insights for targeting SMARCA4 in solid tumors.

*SMARCA4* is currently classified as a lower penetrance gene that may be included as a part of multi-gene testing in the National Comprehensive Cancer Network guideline (26). Additionally, it is not included in “ACMG SF v3.2 list for reporting of secondary findings in clinical exome and genome sequencing” which comprises genes associated with medically actionable conditions (27). The inclusion criteria of this list are based on whether identifying a variant in that gene can lead to preventive or therapeutic interventions. The absence of SMARCA4 from this list reflects the fact that its association with hereditary cancer syndromes has not yet been widely recognized, largely due to the scarcity of reports. Nonetheless, our case contributes a layer of evidence that a germline *SMARCA4* pathogenic variant, may have broader implications in cancer predisposition beyond the currently recognized associations. It also underscores the clinical utility of comprehensive gene panel testing. Enhanced early detection and surveillance strategies through genetic counseling could potentially improve outcomes by facilitating earlier diagnosis and intervention. Especially for young female patients, the pros and cons of fertility preservation options should be thoroughly discussed as part of the treatment or surveillance strategy (28, 29).

While the evidence might not be deemed conclusive, the findings offer encouraging signals that targeting the loss of SMARCA4 may represent an effective approach for treating this particularly aggressive cancer type. With the increasing application of DNA sequencing, it’s expected that the diagnosis of *SMARCA4*-deficient sarcomas will become more frequent in the forthcoming period. Further studies are urgently needed to explore the potential of targeting SMARCA4 deficiency therapeutically. Such research efforts are essential for advancing clinical knowledge and have the potential to significantly improve treatment outcomes as individuals with this genetic alteration. Additionally, to better understand the association between *SMARCA4* variants and extraskeletal Ewing sarcoma, further validation in larger cohorts is required.

## Data Availability

The original contributions presented in the study are included in the article/**Supplementary Material**, further inquiries can be directed to the corresponding author.
