# Supplementary material for: Case report: Extraskeletal Ewing sarcoma with a germline pathogenic variant of SMARCA4
Source: Front Oncol. 2024 Oct 8;14:1422605. doi: 10.3389/fonc.2024.1422605 (PMC11493533; doi:10.3389/fonc.2024.1422605)
Supplement: Supplementary file 1 [file Table1.docx]

S1 Table. A list of 73 genes included in germline next-generation sequencing (NGS) panel

| **Classification** | **Gene name** |
| --- | --- |
| Hereditary NGS panel  (73 genes) | *AIP, APC, ATM, AXIN2, BAP1, BARD1, BMPR1A, BRCA1, BRCA2, BRIP1, CDC73, CDH1, CDK12, CDK4, CDKN1B, CDKN2A, CHEK1, CHEK2, CTNNA1, DICER1, EPCAM, EXT1, EXT2, FANCL, FH, FLCN, GREM1, HOXB13, HRAS, KIT, MAX, MEN1, MET, MLH1, MRE11, MSH2, MSH3, MSH6, MUTYH, NBN, NF1, NF2, NTHL1, PALB2, PMS2, POLD1, POLE, PPP2R2A, PTCH1, PTEN, RAD50, RAD51B, RAD51C, RAD51D, RAD54L, RB1, RET, SDHA, SDHAF2, SDHB, SDHC, SDHD, SMAD4, SMARCA4, SMARCB1,STK11, SUFU, TMEM127, TP53, TSC1, TSC2, VHL, WT1* |
